# Supplementary material for: Inflammation mobilizes copper metabolism to promote colon tumorigenesis via an IL-17-STEAP4-XIAP axis
Source: Nat Commun. 2020 Feb 14;11:900. doi: 10.1038/s41467-020-14698-y (PMC7021685; doi:10.1038/s41467-020-14698-y)
Supplement: Supplementary file 1 — Supplementary Information [file 41467_2020_14698_MOESM1_ESM.pdf]

Inflammation mobilizes copper metabolism to promote colon tumorigenesis via an IL-17-STEAP4-XIAP axis

Liao et al.

Supplementary Figure. 1

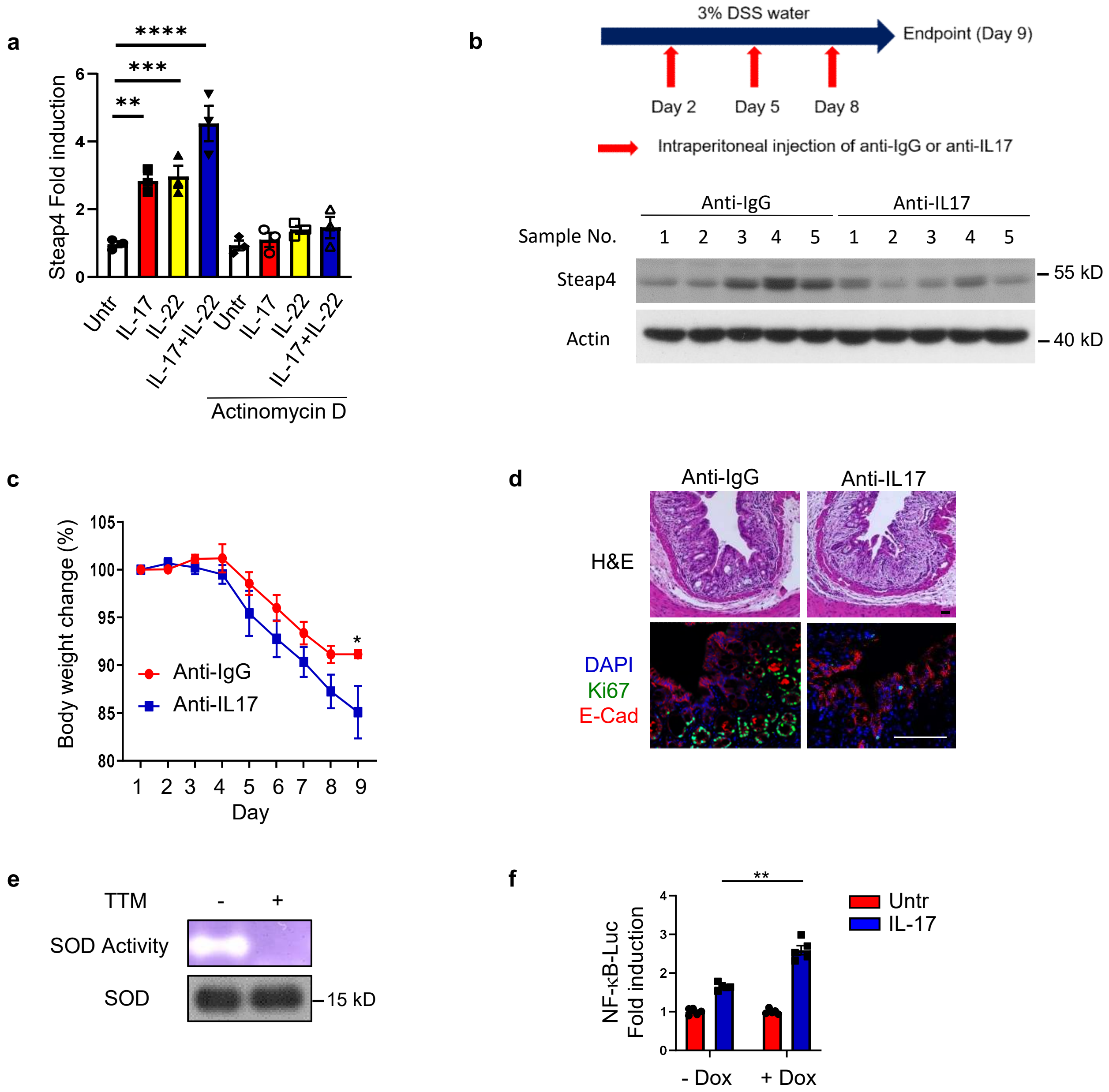

Supplementary Figure. 1 Neutralization of IL-17 attenuated Steap4 induction in DSS colitis model. **a.** Ls174t cells were treated with indicated ligands for 4 hours with or without 5mg/ml Actinomycin D pretreatment for 1 hour. STEAP4 expression was analyzed by RT-PCR. **, P= 0.0012; ***, P=0.0006; ****, P<0.0001 by two-tailed Student's t test. Error bars show S.E.M. of 3 biological replicates. **b.** Western blot analysis for Steap4 expression from mice treated with anti-IgG or anti-IL17 antibody. Mice were put on 3% DSS water for 9 days. Anti-IgG or anti-IL17 were intraperitoneally injected at the indicated time points until endpoint. (n=5 mice) **c.** Weight loss plot for mice from panel (**b**). n=5 mice. *, P=0.0426 by two-tailed Student's t test. **d.** Histology analysis for DSS treated mice with and without IL-17 neutralization. Representative H&E staining and immunofluorescence staining (Ki67 and E-cadherin) were shown. Scale bar, 100μm. **e.** In-gel SOD activity assay of colon organoids treated or untreated with TTM. **f.** NF-κB luciferase assay of untreated and IL-17-treated STEAP4-inducible Ls174t cells pre-treated with or without Dox. **, p=0.0079 by two-tailed Student's t test(n=5 biologically independent cell culture). *In vivo* data were not pooled. All experiments were repeated twice and yielded consistent results. All data were presented as mean ± SEM.

Supplementary Figure. 2

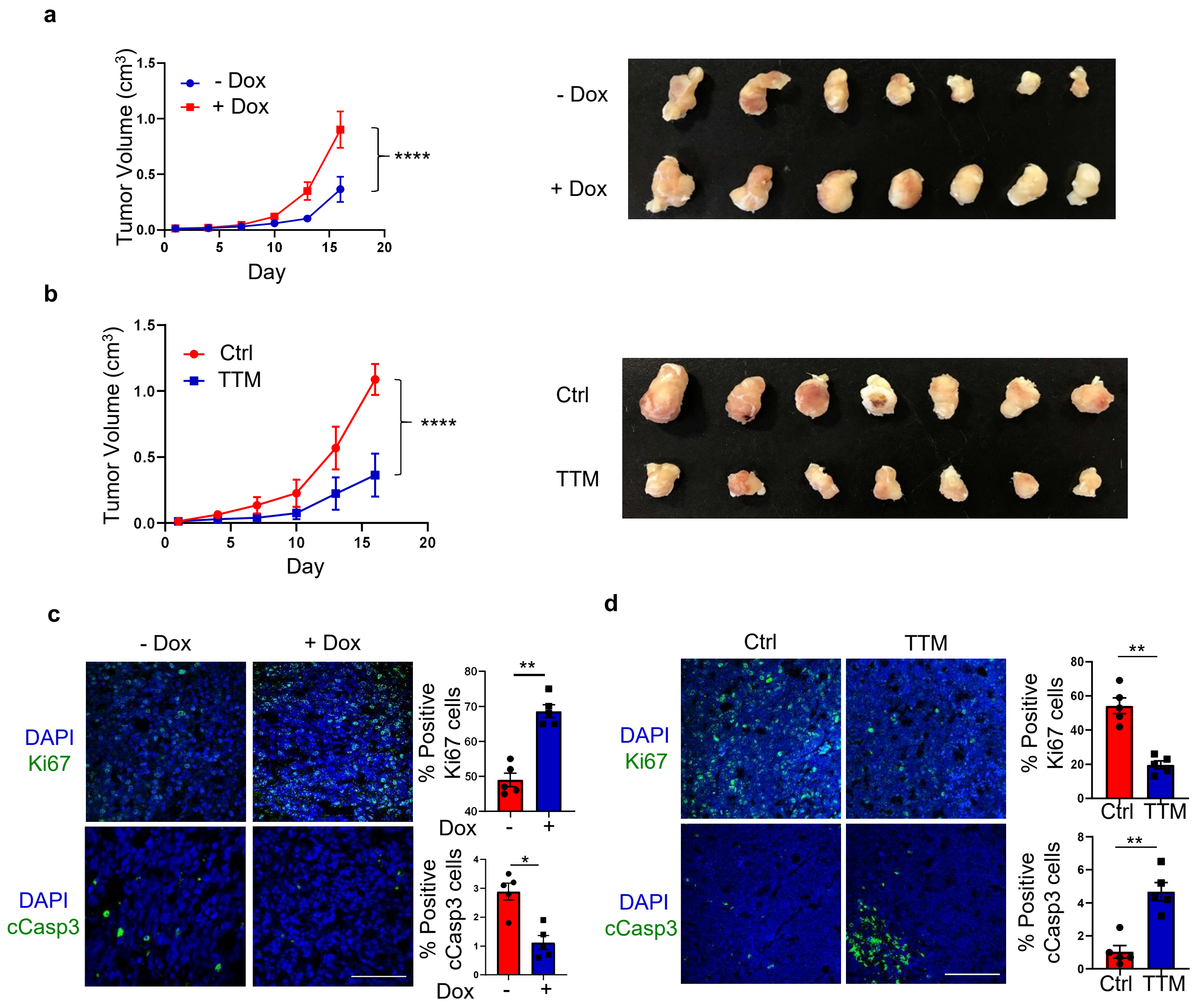

Supplementary Figure. 2 STEAP4-induced copper uptake promoted xenografted tumor growth **a.** Growth kinetics and macroscopic image of harvested flank xenograft tumors derived STEAP4 inducible Ls174t cells pretreated with or without Dox to induce Steap4 overexpression. Data are shown as mean ± SEM. ****, P<0.0001 by two way ANOVA analysis followed by Sidak's multiple comparisons test. (n=7 mice) **b** Growth kinetics and macroscopic image of harvested flank xenograft tumors derived Dox-primed STEAP4 inducible cells treated with TTM (10 mg/kg, once per week) or saline (Ctrl). Data are shown as mean ± SEM. ****, P<0.0001 by two way ANOVA analysis followed by Sidak's multiple comparisons test for treatment group. (n=7 mice) **c.** Immunofluorescence staining of Ki67 and cleaved Caspase-3 for implanted tumor tissue from panel (**a**). Scale bar,100μm. Data are shown as mean ± SEM. *, P=0.0219; **, P=0.0034 by two-tailed Student's t test (n=7 tumors). **d.** Histology analysis of Ki67 and cleaved Caspase-3 for tumor tissue from panel (**b**). Quantification was performed based on five high magnification fields from each sample and data were presented as mean ± SEM. **, P= 0.0021 for Ki67; **, P=0.0047 for cCasp3 by two-tailed *Student*'s t test. Scale bar represents 100μm.

Supplementary Figure. 3

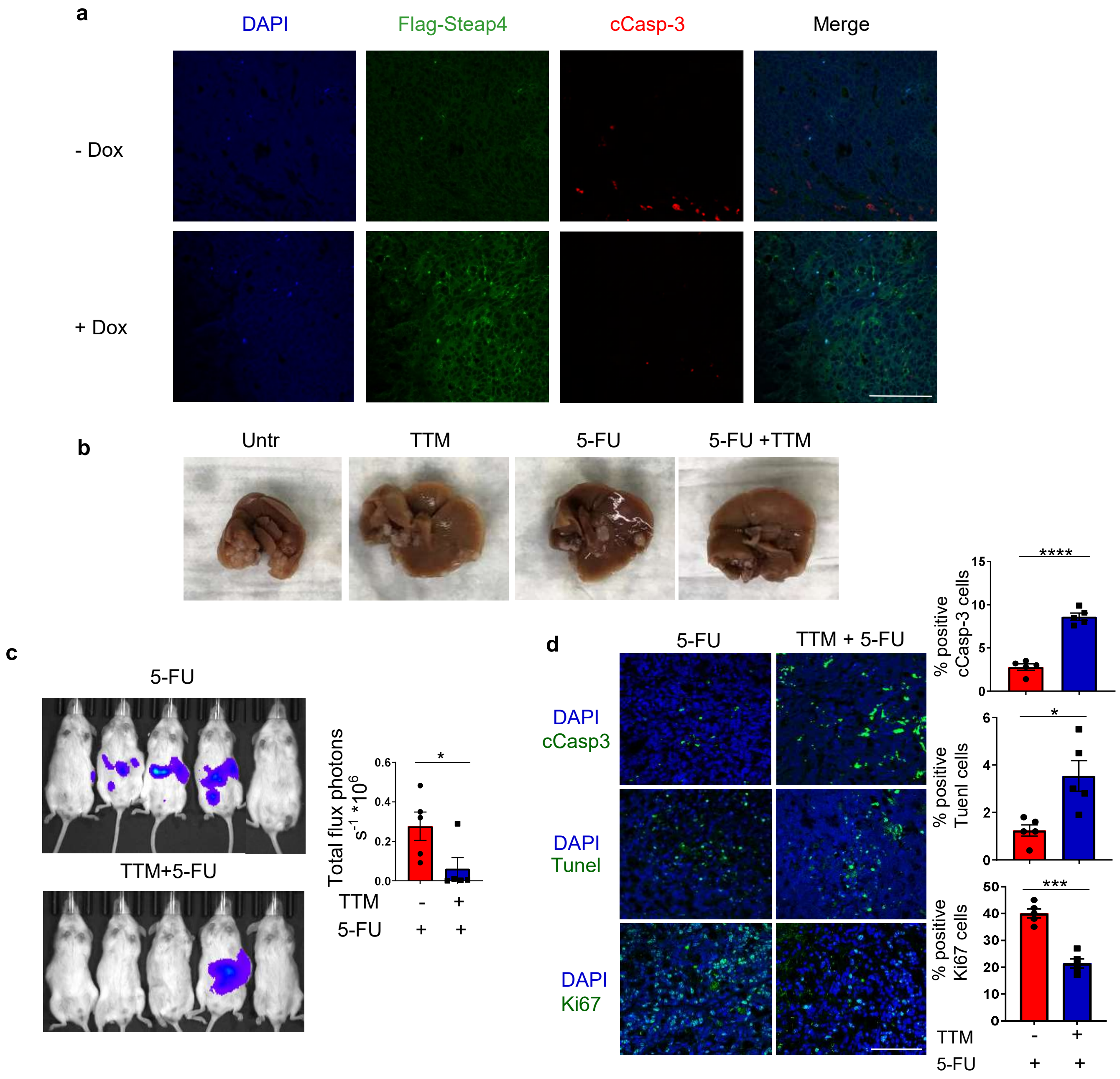

Supplementary Figure. 3 Copper chelation abolished STEAP4-mediated chemoresistance

a. Immunofluorescent staining of metastatic tumor nodules. Cleaved caspase-3 (cCasp3) and Flag were stained. Scale bar represents 100μm. **b.** Macroscopic view of liver metastasis tumors. **c.** In vivo bioluminescence measurement and quantification of mice bearing STEAP4 overexpression tumor treated with 5-FU alone or with copper chelation adjuvant therapy. Data were presented as mean ± SEM. *, P=0.047 by two-tailed Student's t test (n=5 mice). **d.** Immunofluorescent staining of metastatic tumor nodules from mice treated with indicated agents. Quantification was performed based on 5 tumors under high magnification and data were presented as mean ± SEM of 5 biological replicates. *, P=0.0101; ***, P=0.0009; ****, P<0.0001 by two-tailed Student's t test. Scale bar ,100μm.

Supplementary Figure. 4

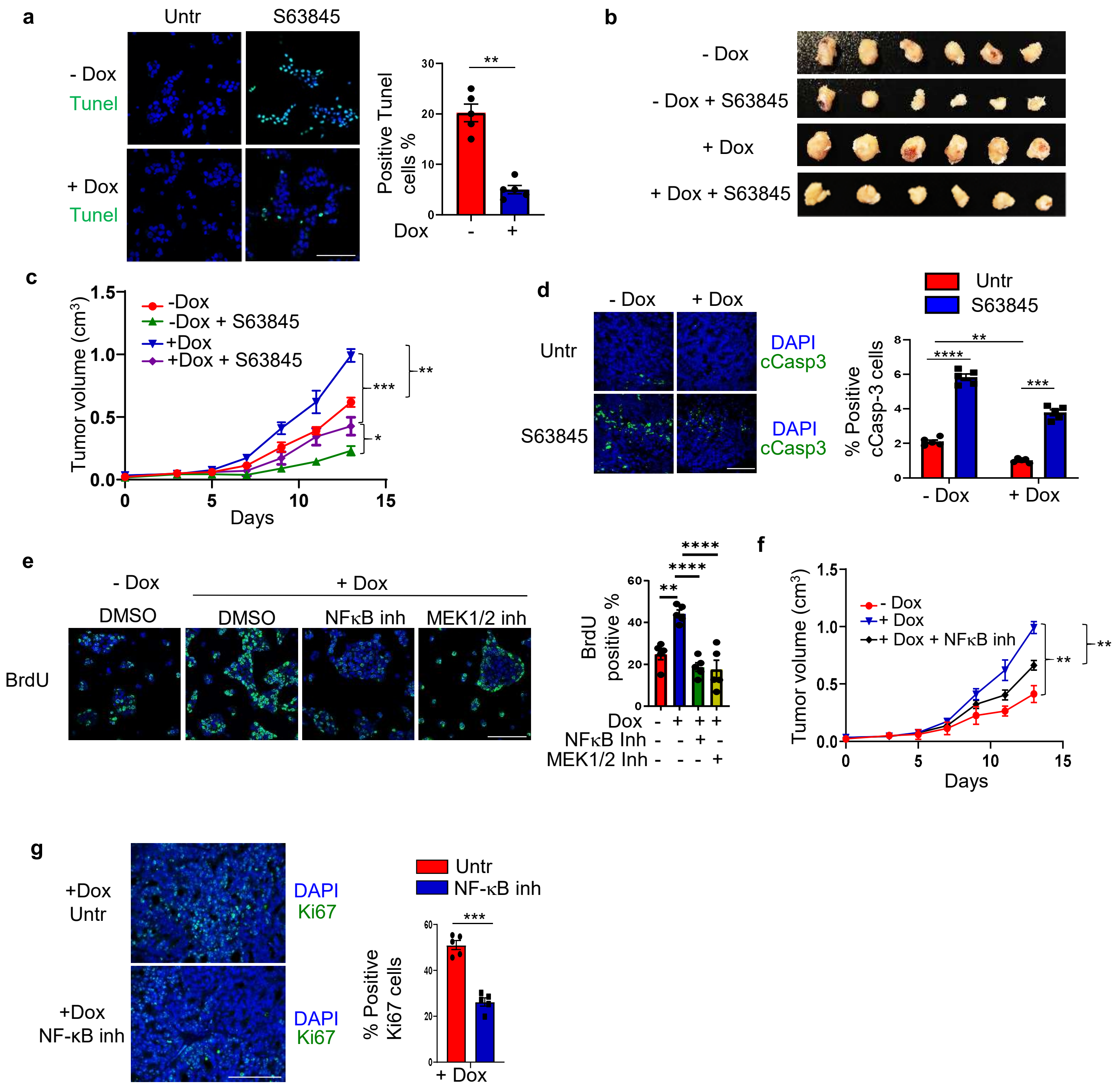

Supplementary Figure. 4 The impact of STEAP4 expression on tumor response to inhibitors. a TUNEL assay of S63845-treated (8hrs) STEAP4-inducible Ls174t cells pre-treated with/without Dox in the presence of IL-17. Quantification were performed based on 5 independent wells and data were presented as mean ± SEM. **, P=0.0027 by two-tailed t-test. Scale bar, 100µm. **b.** Macroscopic tumor image from mice injected with or without Dox induced STEAP4 inducible Ls174t cells. Mice were treated with S63845 every other day until endpoint (n=6 mice). **c** Tumor growth kinetics for panel (**b**). Data were presented as mean ± SEM. *, P= 0.0165; **, P=0.004; ***, P= 0.0007 by two-way ANOVA analysis followed by Tukey's test. **d** Immunohistochemistry analysis of activated caspase 3 in tumor tissue from mice with indicated treatment. Quantification was performed based on five high magnification fields from each sample and data were presented as mean ± SEM, n=6 biological replicates; **, P=0.0055, ***, P=0.002, ****, P<0.0001 by two-way ANOVA followed by Sidak's test. Scale bar, 100µm. **e.** STEAP4-expressing and control Ls174t cells with indicated treatment (NFκB inhibitor (NFκB inh): BMS-345541 50µM; MEK1/2 inhibitor (MEK1/2 inh): trametinib 10nM) were primed with IL-17 (12hrs) and then pulsed with BrdU (10 µM) for 4 hours, followed by immunofluorescent staining for BrdU. Quantification was performed based on high magnification fields from 5 independent wells and data were presented as mean ± SEM. **, P=0.0012; ****, P< 0.0001 by two-tailed student's t test. Scale bar, 100µm. **f.** Growth kinetics of xenografted tumor receiving indicated treatments (NFkB inhibitor = BMS-345541), n=6. Data were presented as mean ± SEM. **, P= 0.0024 (- Dox vs. + Dox) and 0.0027 (+ Dox vs. +Dox+NF-κB inh) by two-way ANOVA followed by Tukey's test. **g.** Immunohistochemistry analysis of Ki67 in tumor tissue from mice with indicated treatment. Quantification was performed based on five high magnification fields from each sample and data were presented as mean ± SEM of 4 biological replicates. Data are shown as mean ± SEM. ***, p=0.0009 by two-tailed t test. Scale bar, 100µm. Experiment in a and e was repeated 3 times with consistent results. The result of the data were collected from one experiment.

Supplementary Figure. 5

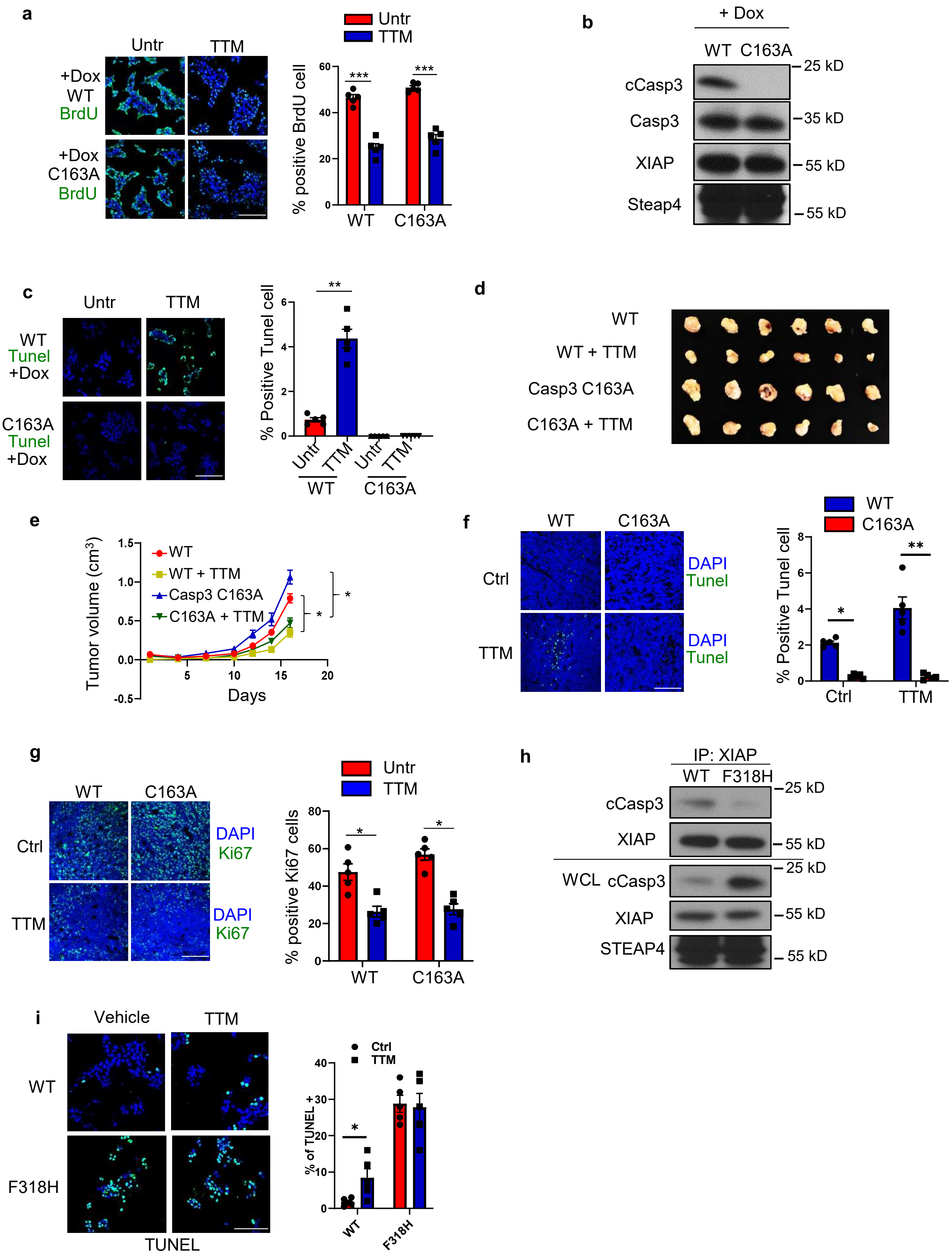

Supplementary Figure. 5 STEAP4-induced copper uptake increased cell proliferation. **a.** IL-17-stimulated STEAP4-expressing Ls174t clones carrying wild-type or C163A caspase 3 were treated with TTM for 6 hours. Treated cells were pulsed with BrdU for 4 hours and then subjected to immunofluorescent staining for BrdU. Quantification was performed based on high magnification fields from 5 independent wells. ***, P= 0.0002 by two-tailed Student's t test. Scale bar, 100μm. **b.** Western blot validation of wild-type and mutant caspase 3 expression. **c.** TUNEL assay of IL-17-stimulated STEAP4-inducible Ls174t clones expressing wild-type or C163A caspase 3 in the presence or absence of TTM treatment. Quantification were performed based on 5 independent cell cultures. **, P= 0.0021 by two-sided Student's t test. Scale bar, 100μm. **d.** Macroscopic tumor image from mice injected STEAP4-expressing Ls174t cells with wild-type or C163A caspase 3. n=6 tumors. **e.** Tumor growth kinetics for panel (**c**). Data were presented as mean ± SEM, n=6 mice. *, P= 0.0182 (WT vs. WT + TTM); *, P= 0.0106 (C163A vs. C163A + TTM) by two-way ANOVA followed by Tukey's multiple tests. **f-g.** TUNEL assay (**f**) and immunohistochemistry analysis of Ki67 (**g**) in tumor tissue from mice with indicated treatment. Quantification were performed based on high magnification fields from 5 independent wells. *, P= 0.0333, **, P= 0.0025 for panel **f;** *, P=0.0368 for WT cell and P=0.0115 for C163A cell in panel **g** using two-tailed Student's t test. Scale bar, 100μm. **h.** STEAP4-expressing Ls174t cells with either wild-type or F381H caspase 3 were stimulated with IL-17 for 24 hours. Cells were lysed and lysates were immunoprecipitated with anti-XIAP followed by western blot analysis of indicated proteins. **i.** TUNEL assay of IL-17-stimulated STEAP4-expressing Ls174t cells with either wild-type or F318H caspase 3 treated or untreated with TTM.. Quantification were performed based on high magnification fields from 5 independent wells. *, P=0.0256 by two-tailed Student's t test. Scale bar, 100μm. All data were presented as mean ± SEM. Experiments in a-c and h-I were repeated twice with consistent results.

Supplementary Figure. 6

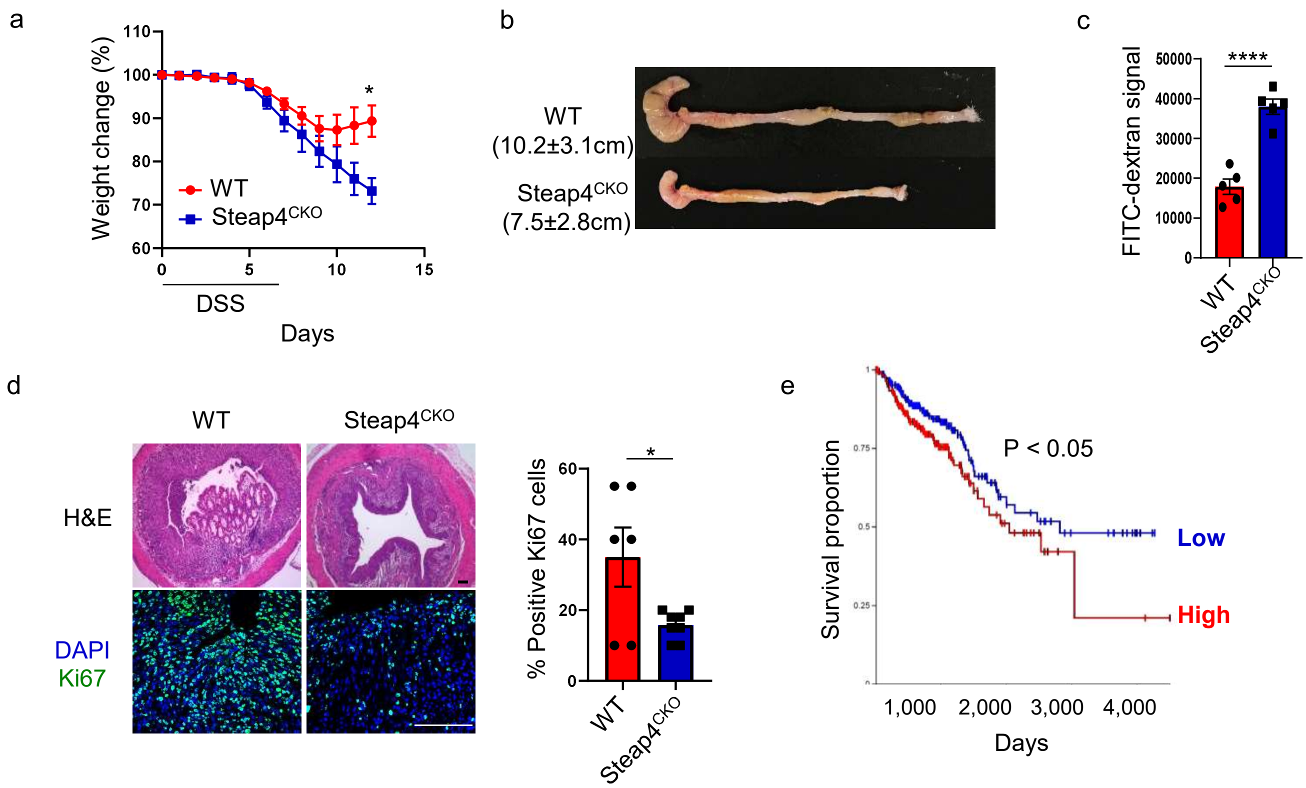

Supplementary Figure. 6 The role of STEAP4 in DSS-induced colitis. Colonic epithelial cell specific STEAP4 knockout STEAP4^{CKO} and littermate control mice were subjected to acute DSS challenge with 3 % DSS water for 7 days followed by 5 days of recovery on regular water, n=8 mice. **a.** Weight loss presented as a percentage of original body weight. *, p=0.0157 by two-tailed Student's t test. **b.** Macroscopic view of colon length. **c.** Gut permeability assayed with dextran-FITC gavage (0.5g/kg) followed by analysis of serum FITC signal 6 hours later. ****, p<0.0001 by two-tailed Student's t test (n=5 mice). **d.** Immunohistochemistry analysis of Ki67 in colon tissues harvested 5 days after DSS challenge. Quantification was performed based on 6 high magnification field views. *, p=0.0222 by unpaired two-tailed Student's t test. Scale bar, 100μm. **e.** Kaplan-Meier analysis of colorectal cancer patients in TCGA dataset stratified by median STEAP4 expression (n=361 cases). p<0.05 by log-rank test.

Supplementary Figure. 7

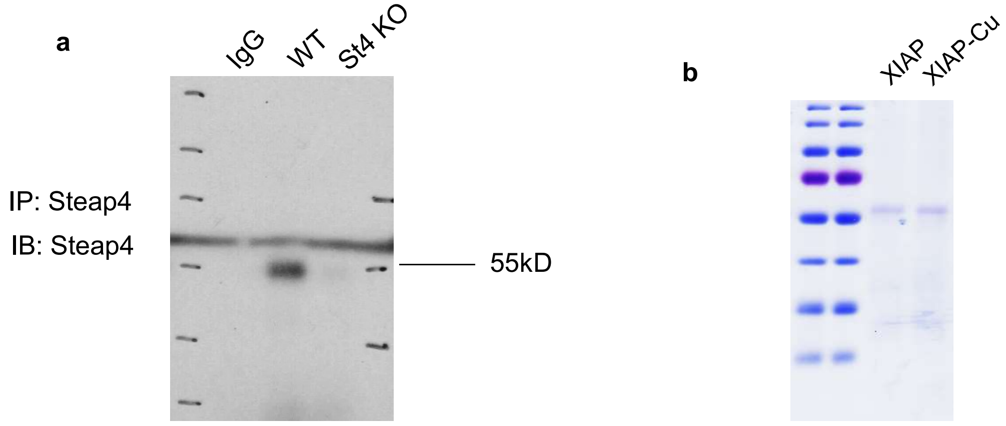

Supplementary Figure. 7 Control data for cell line and protein purification. a. Validation of STEAP4 knockout Ls174t cells. **b.** Coomassie Blue staining of purified recombinant XIAP resolved on SDS-PAGE.

Supplementary Figure. 8

Fig 1a.

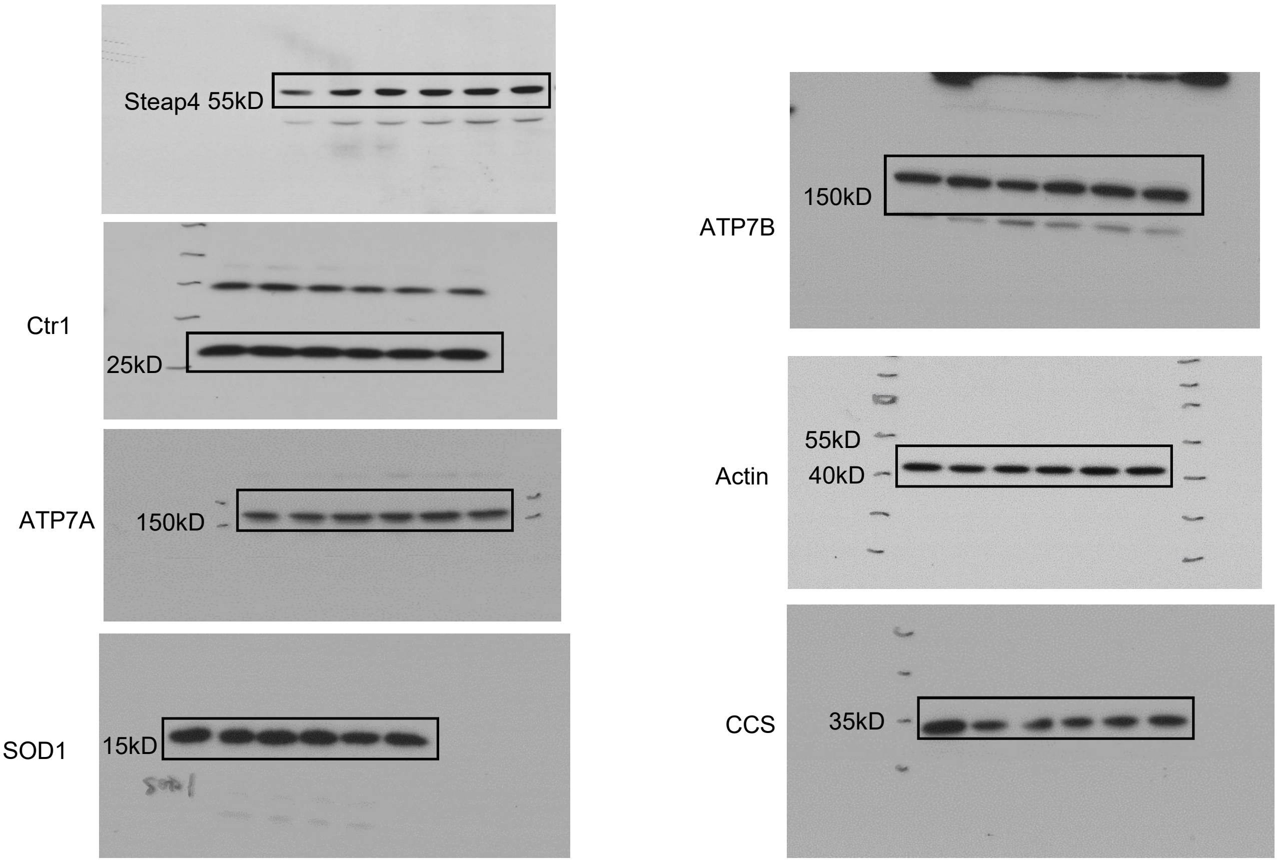

Fig 1c.

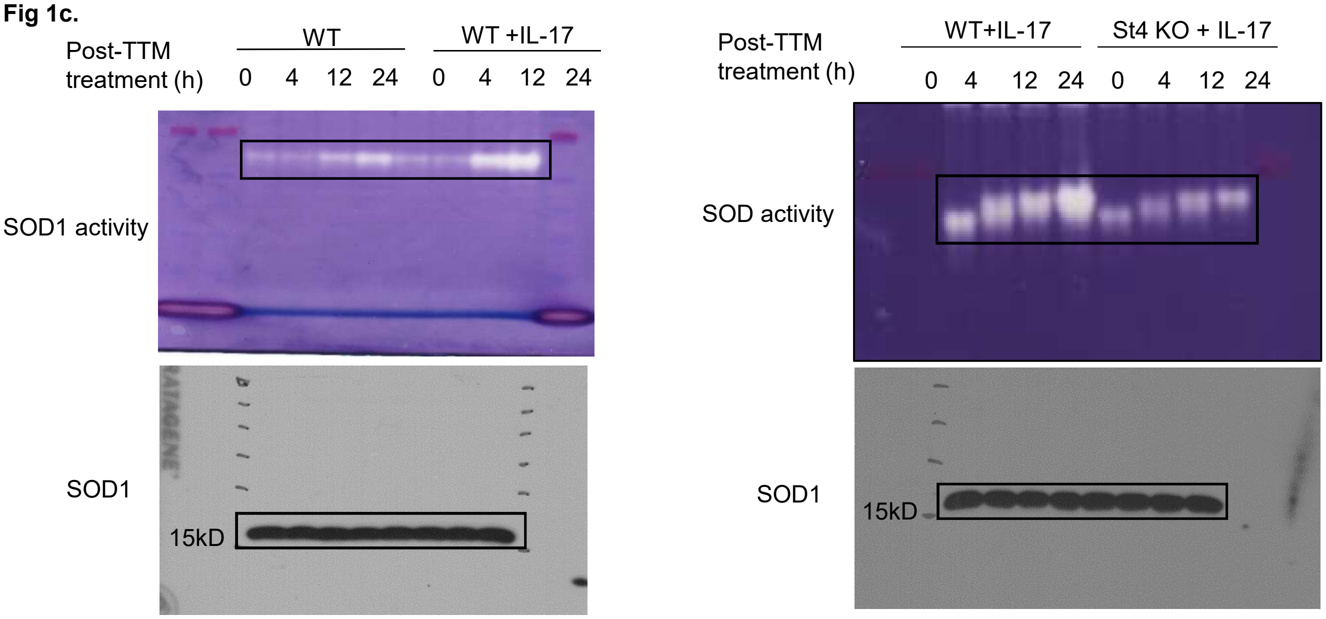

Fig 1g.

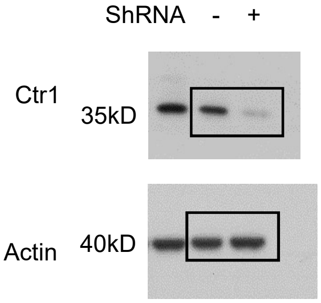

Uncropped blots for Figure 2.

Fig 2a.

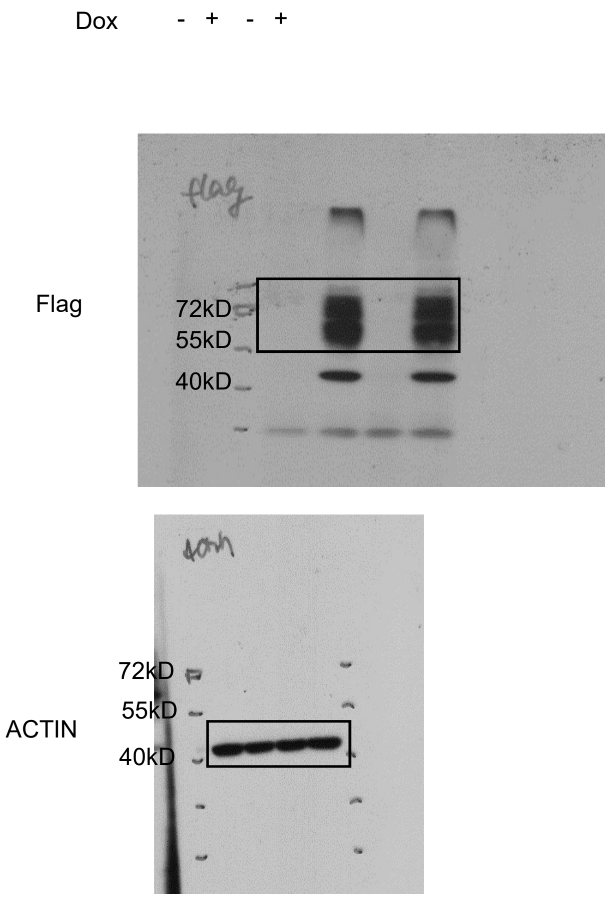

Fig 2c.

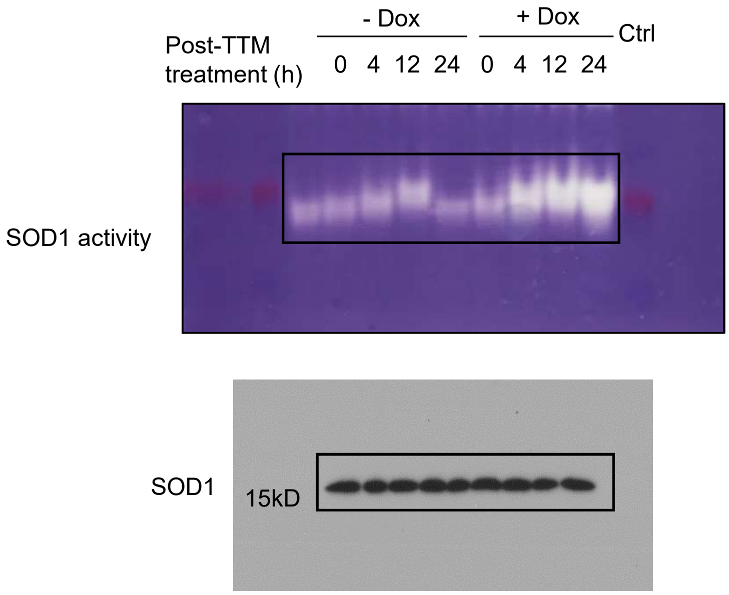

Uncropped blots for Figure 3.

Fig 3a.

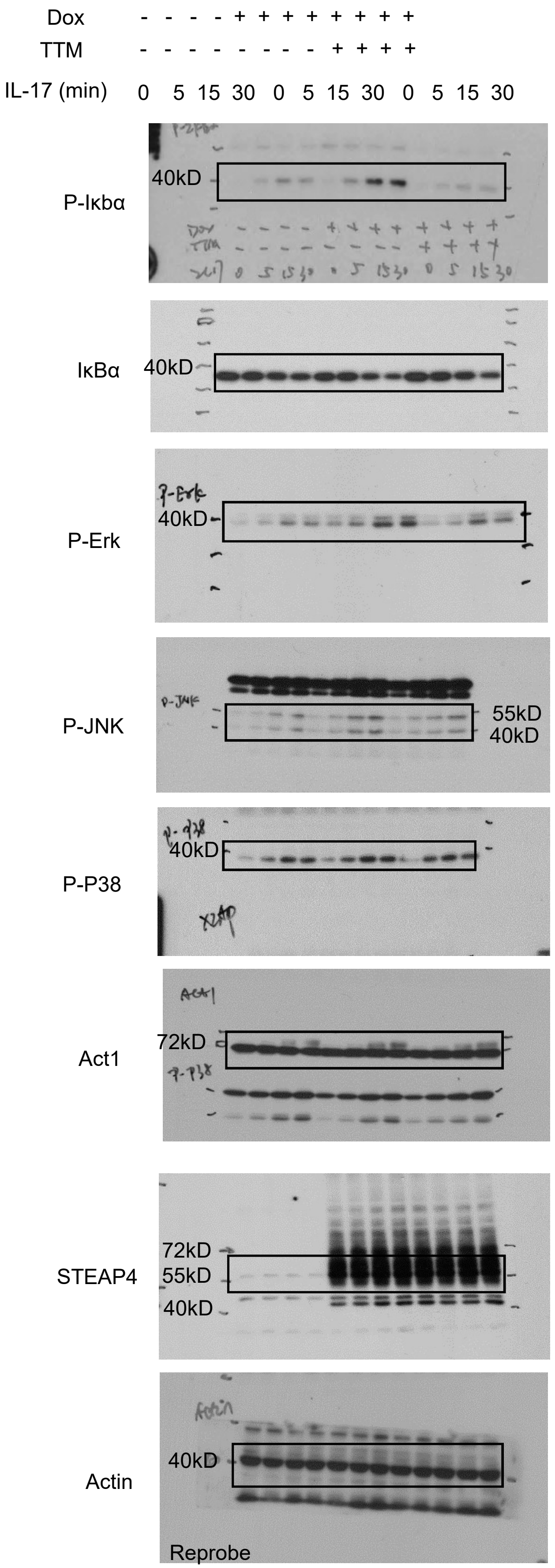

Fig 3b.

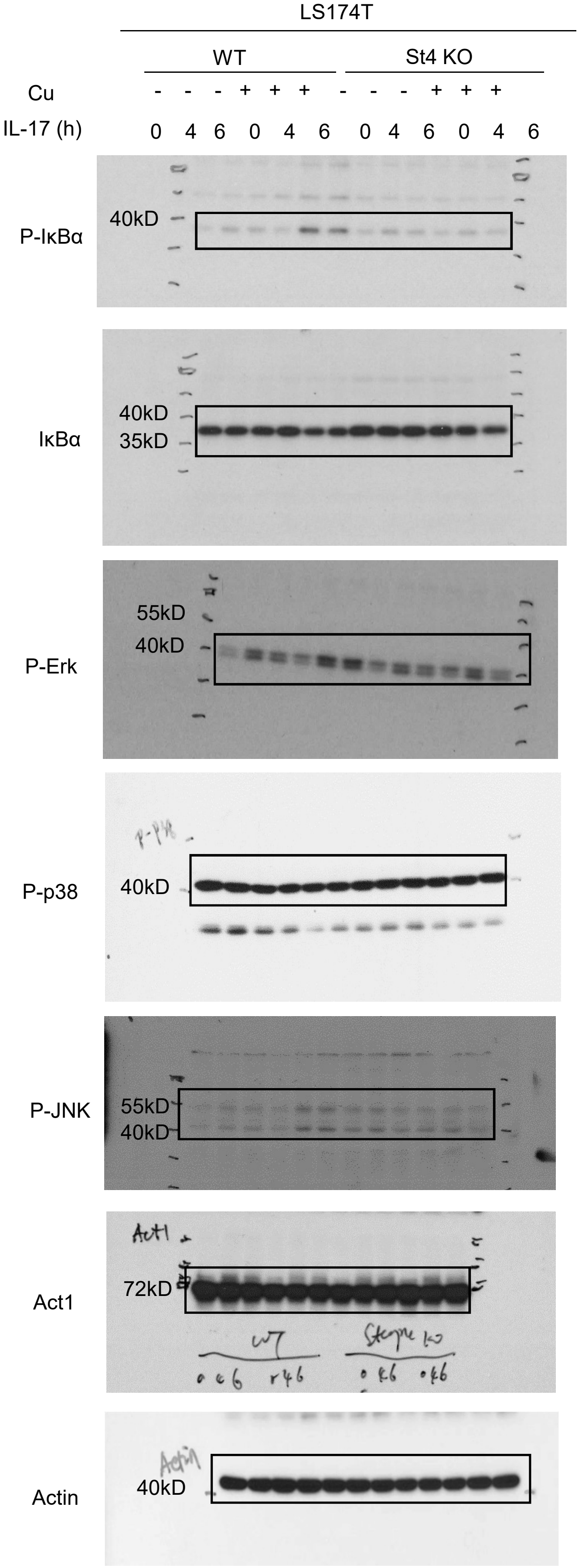

Uncropped blots for Figure 3c

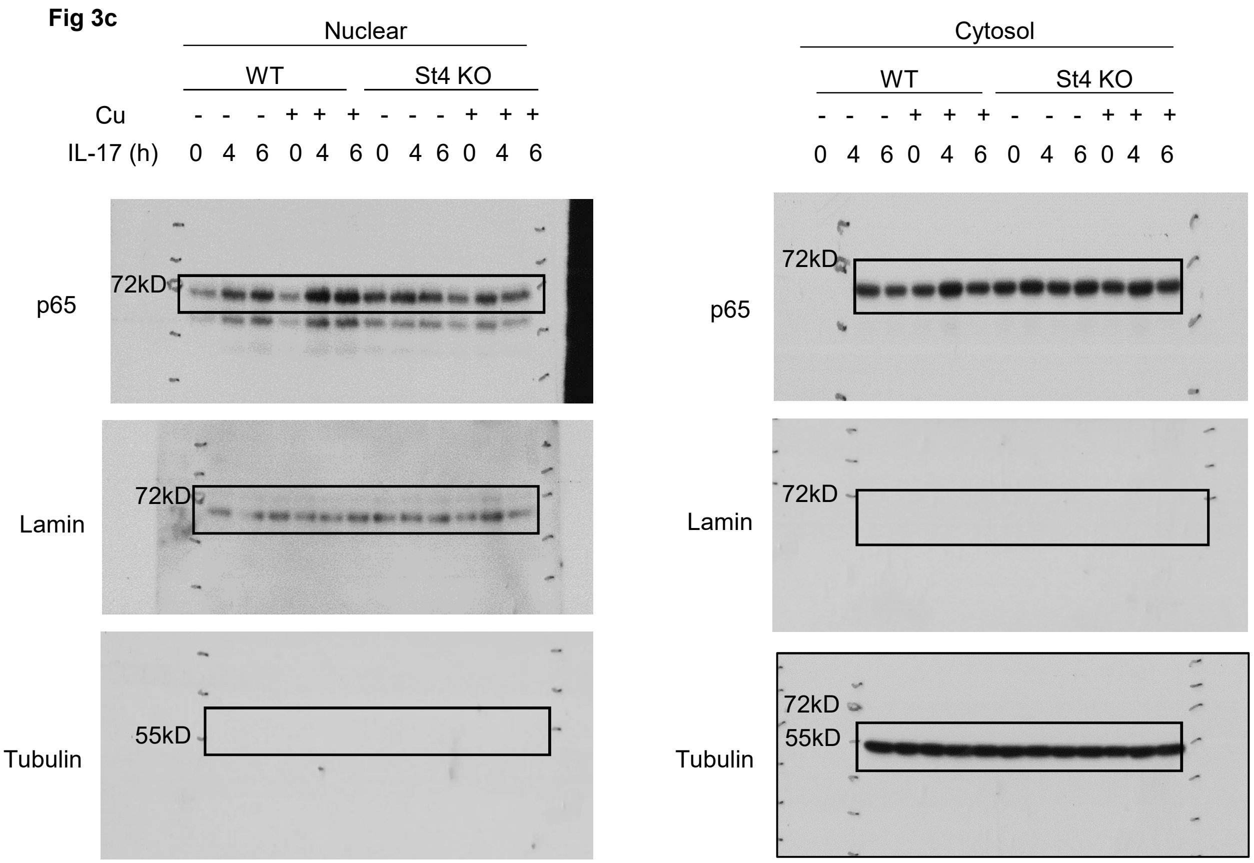

Uncropped blots for Figure 3e

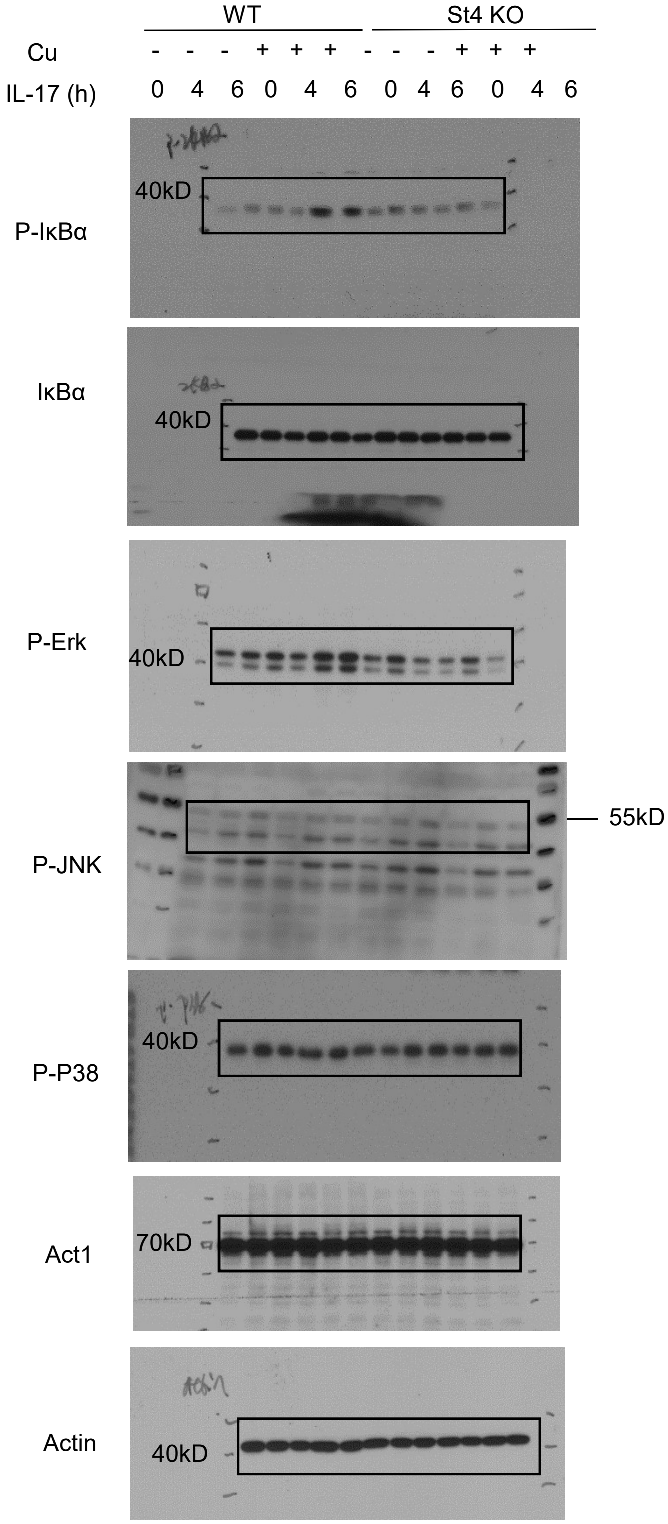

Uncropped blots for Figure 3f

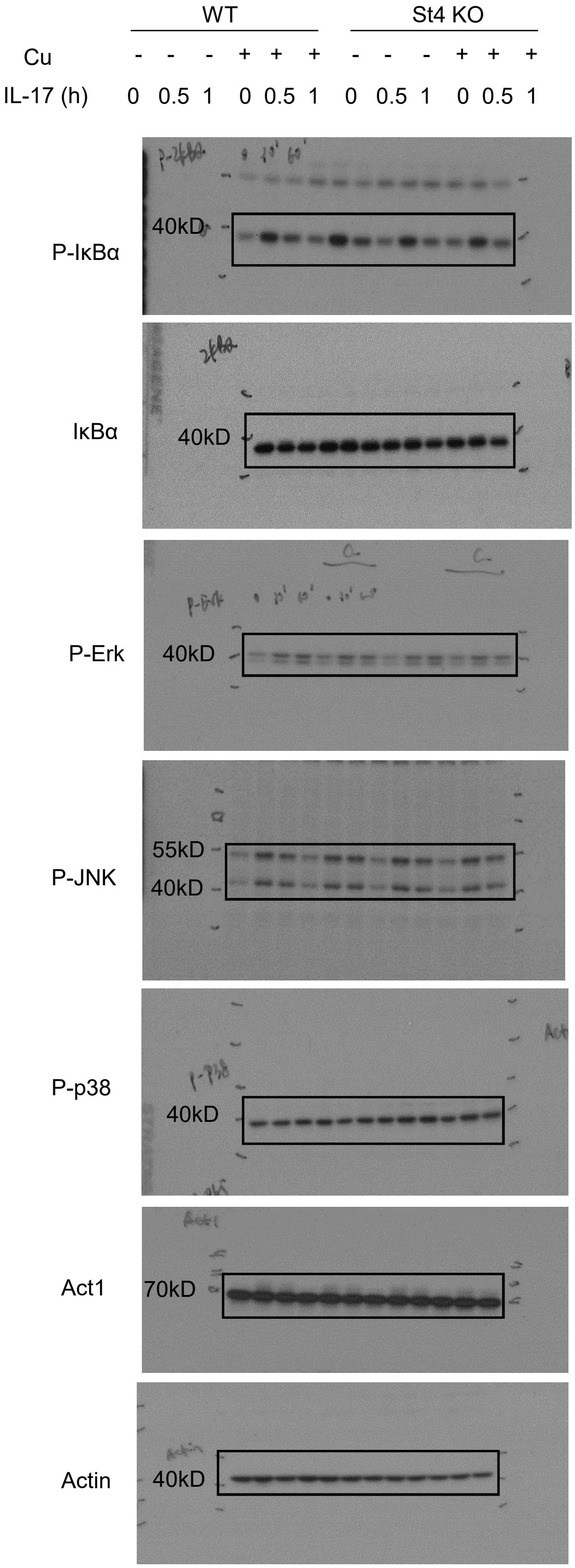

Uncropped blots for Figure 3g.

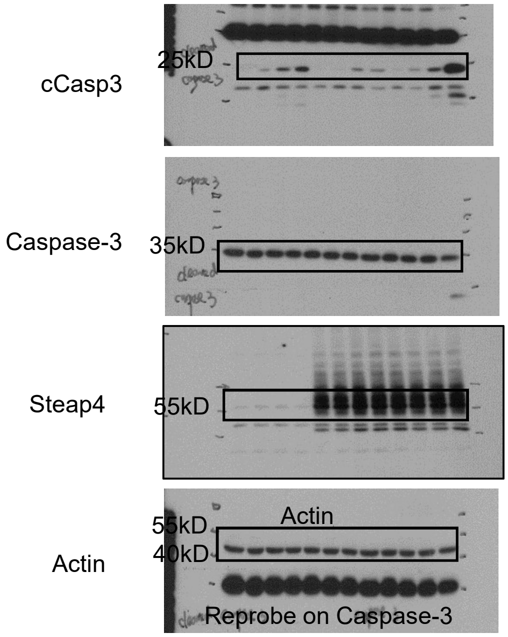

Uncropped blots for Figure 3h.

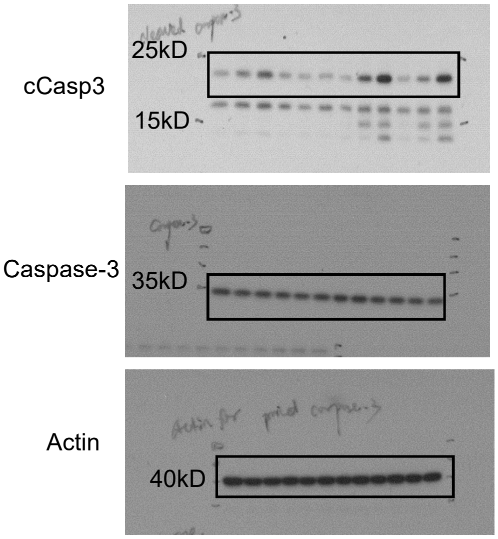

Uncropped blots for Figure 4

Figure 4a.

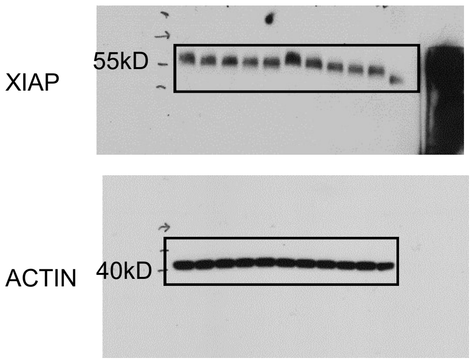

Fig 4b

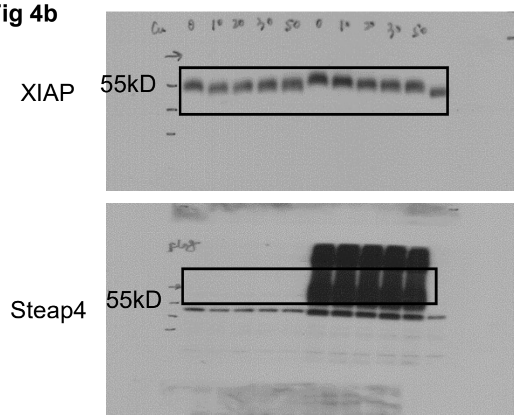

Fig 4c.

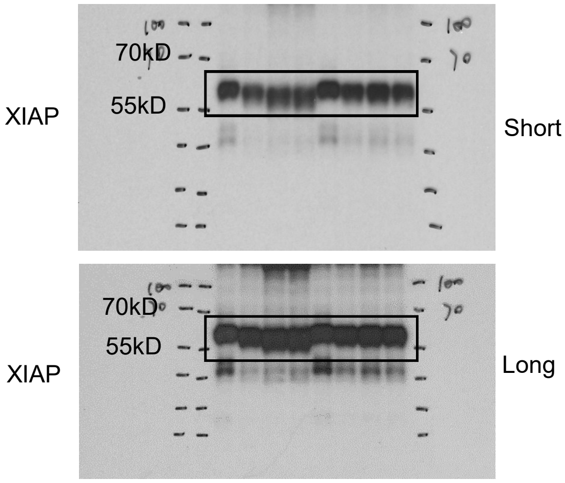

Fig 4d.

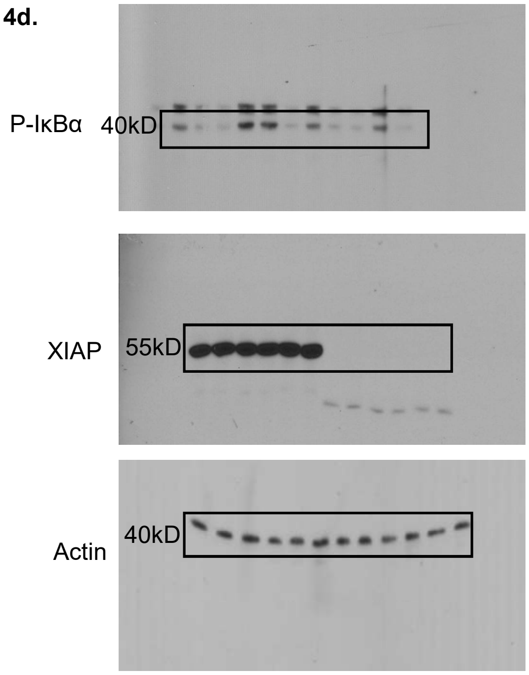

Uncropped blots for Figure 4.

Fig 4e.

cCasp3 25kD

Caspase-3 35kD

XIAP 55kD

Actin 40kD

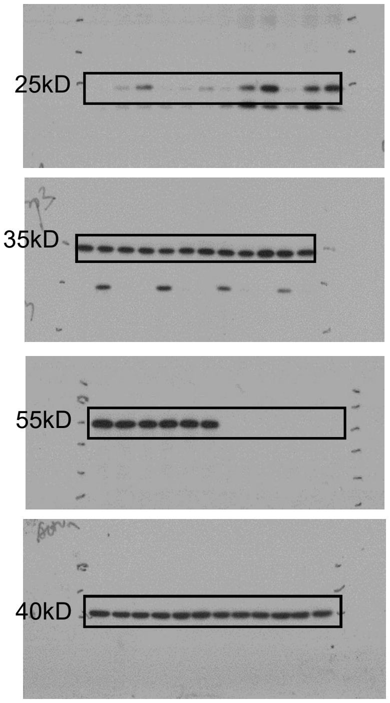

Fig 4f.

+E1, E2, K48-Ub

XIAP	+	-	+	-
XIAP-Cu	-	-	-	+
cCasp3	-	+	+	+

cCasp3 40kD 35kD 25kD

XIAP 55kD

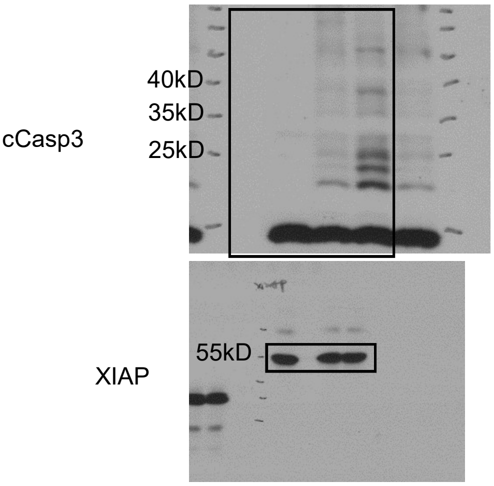

Fig 4g.

SOD1 activity

SOD1 15kD

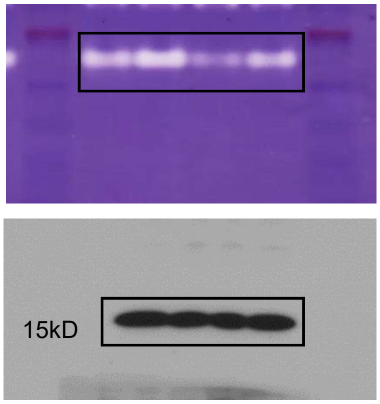

Uncropped blots for Figure 4

Fig 4h.

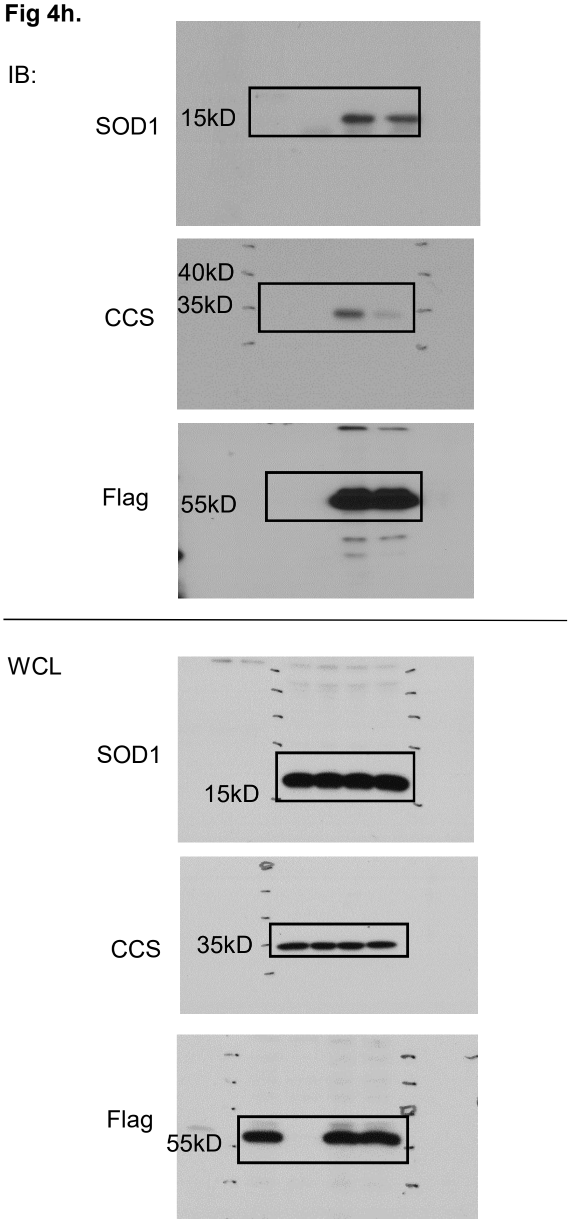

Fig 4i.

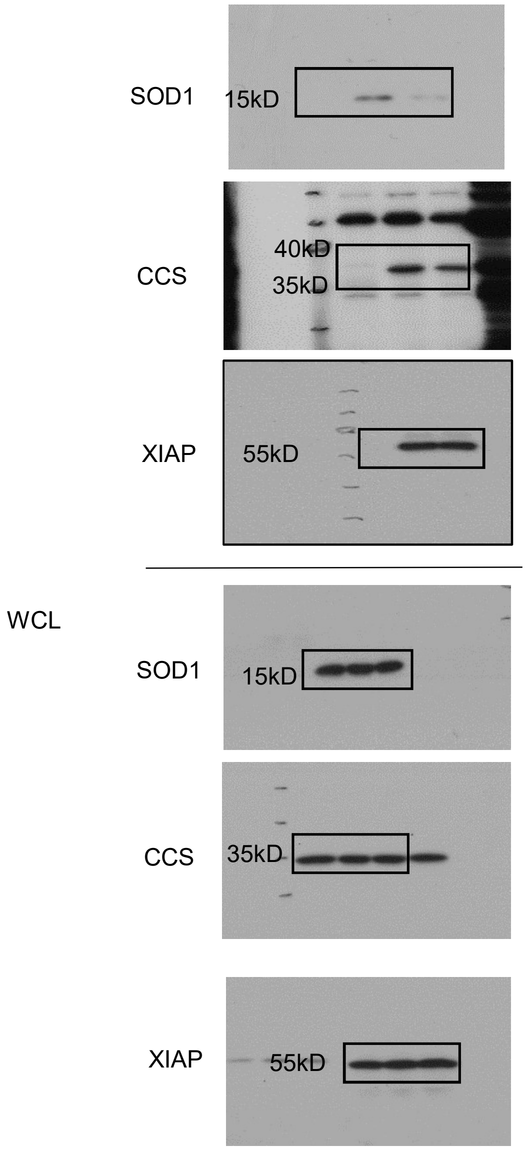

Uncropped blots for Figure 5

Fig 5a.

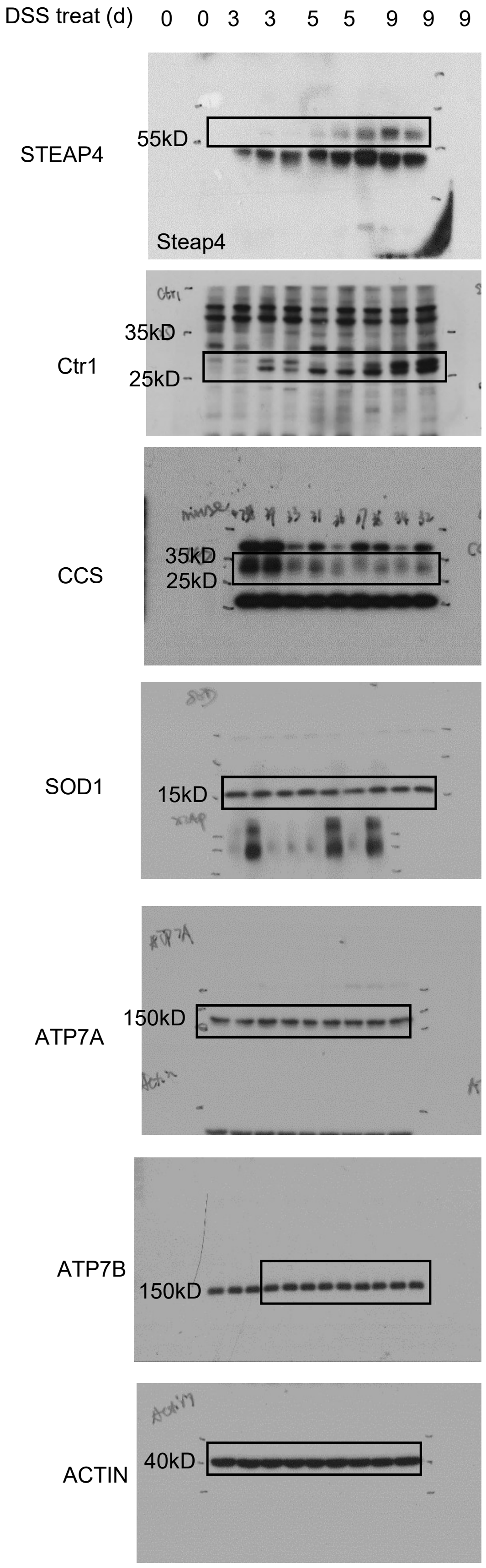

Fig 5b.

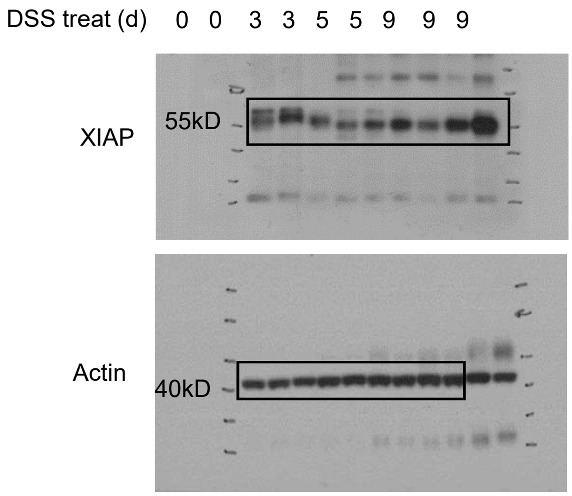

Uncropped blots for Figure 6

Fig 6d.

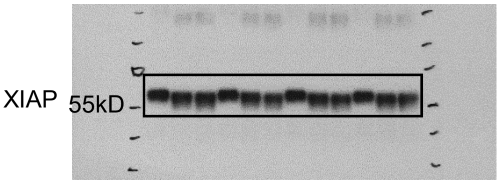

Fig 6g.

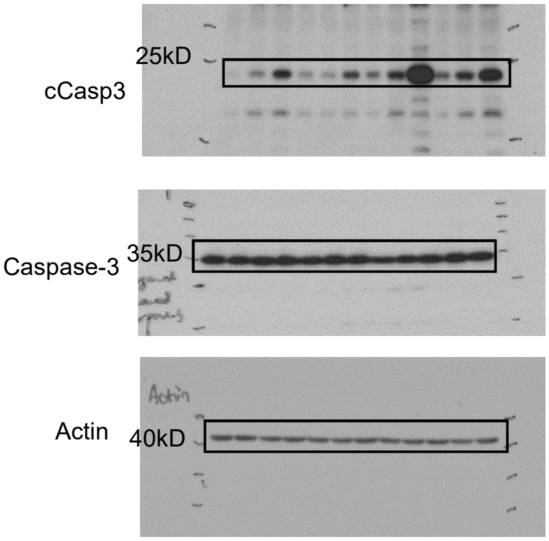

Fig 6h.

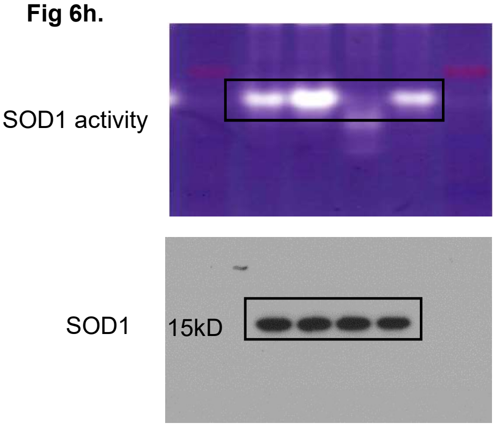

Fig 6f.

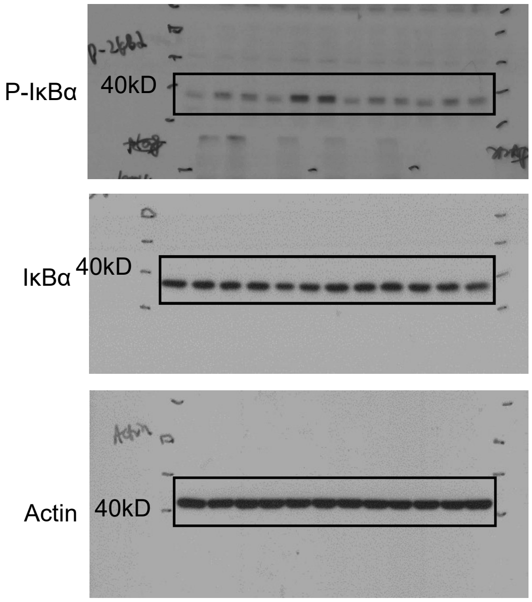

Fig 6e.

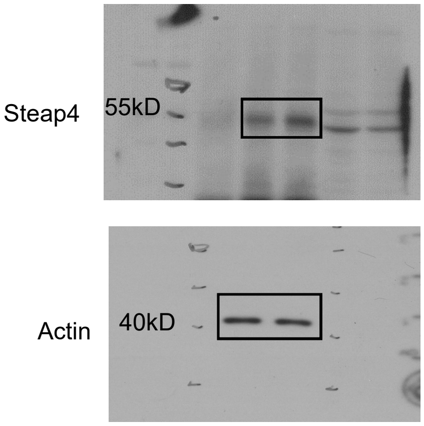

Uncropped blots for Supplementary Figure 1

Supple Fig 1b.

Anti-IgG Anti-IL17

Sample No. 1 2 3 4 5 1 2 3 4

Steap4

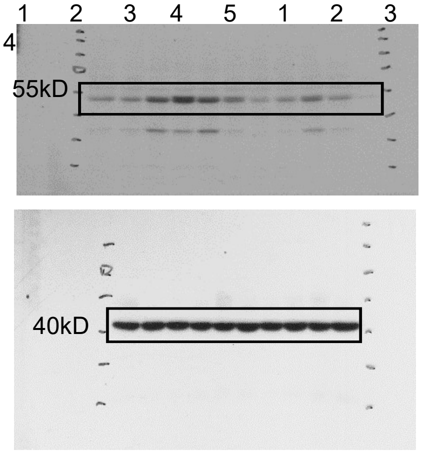

Actin

Supple Fig 1e

TTM - +

SOD Activity

SOD1

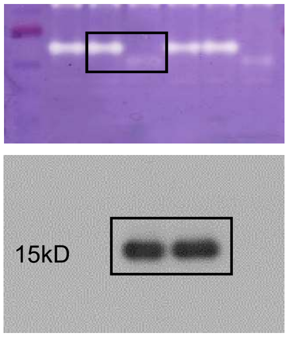

Uncropped blots for Supplementary Figure. 5

5b.

+ Dox

WT C163A

cCasp3

Casp3

XIAP

Steap4

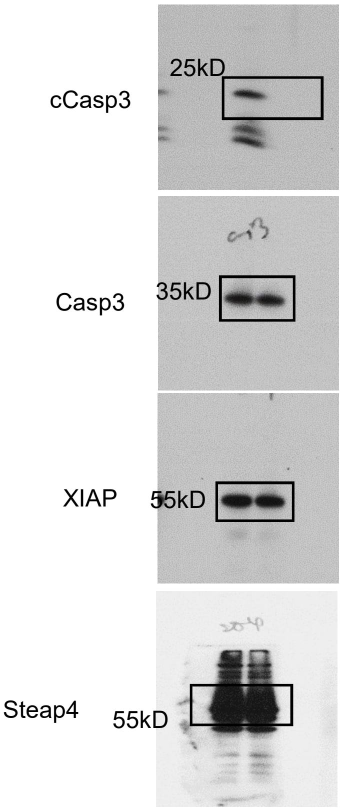

55kD

5h.

IP: XIAP

WT F318h

cCasp3

XIAP

cCasp3

XIAP

Steap4

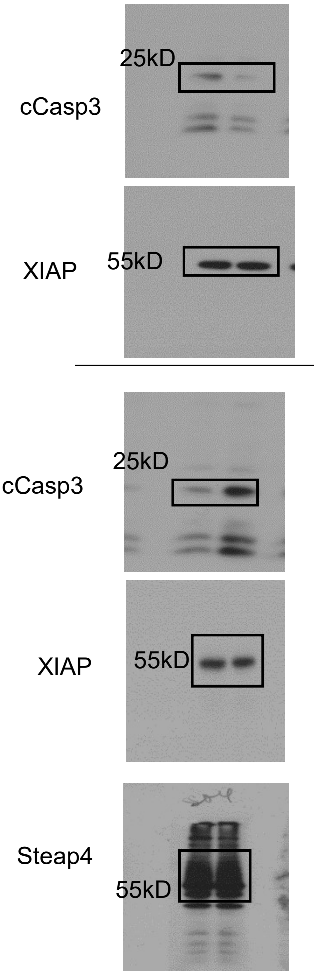

Uncropped blots for Supplementary Figure. 7

7a.

IgG WT St4 KO

IP: Steap4

IB: Steap4

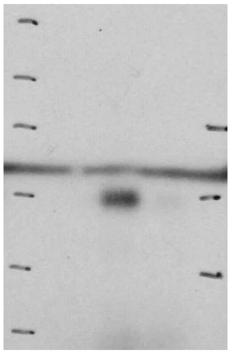

72kD

55kD

7b.

XIAP XIAP-Cu

72kD

55kD

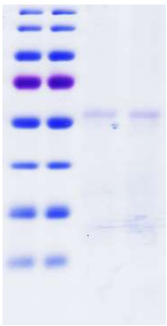

Supplementary Figure 8. Uncropped gels and blots.
Uncropped gels and blots are shown in the order they are presented in the figures.
